# Up-Regulation of ENO1 by HIF-1α in Retinal Pigment Epithelial Cells after Hypoxic Challenge Is Not Involved in the Regulation of VEGF Secretion

**DOI:** 10.1371/journal.pone.0147961

**Published:** 2016-02-16

**Authors:** Feihui Zheng, Wai-Chi Jang, Frederic K. C. Fung, Amy C. Y. Lo, Ian Y. H. Wong

**Affiliations:** Department of Ophthalmology, Li Ka Shing Faculty of Medicine, The University of Hong Kong, Hong Kong, China; University of Nebraska Medical Center, UNITED STATES

## Abstract

**Purpose:**

Alpha-enolase (ENO1), a major glycolytic enzyme, is reported to be over-expressed in various cancer tissues. It has been demonstrated to be regulated by the Hypoxia-inducible factor 1-α (HIF-1α), a crucial transcriptional factor implicated in tumor progression and cancer angiogenesis. Choroidal neovascularization (CNV), which is a leading cause of severe vision loss caused by newly formed blood vessels in the choroid, is also engendered by hypoxic stress. In this report, we investigated the expression of ENO1 and the effects of its down-regulation upon cobalt (II) chloride-induced hypoxia in retinal pigment epithelial cells, identified as the primary source of ocular angiogenic factors.

**Methods:**

HIF-1α-diminished retinal pigment epithelial cells were generated by small interfering RNA (siRNA) technology in ARPE-19 cells, a human retinal pigment epithelial cell line. Both normal and HIF-1α-diminished ARPE-19 cells were then subjected to hypoxic challenge using cobalt (II) chloride (CoCl_2_) or anaerobic chamber. The relation between ENO1 expression and vascular endothelial growth factor (VEGF) secretion by retinal pigment epithelial cells were examined. Protein levels of HIF-1α and ENO1 were analyzed using Western Blot, while VEGF secretion was essayed by enzyme-linked immunosorbent assay (ELISA). Cytotoxicity after hypoxia was detected by Lactate Dehydrogenase (LDH) Assay.

**Results:**

Upon 24 hr of CoCl_2_-induced hypoxia, the expression levels of ENO1 and VEGF were increased along with HIF-1α in ARPE-19 cells, both of which can in turn be down-regulated by HIF-1α siRNA application. However, knockdown of ENO1 alone or together with HIF-1α did not help suppress VEGF secretion in hypoxic ARPE-19 cells.

**Conclusion:**

ENO1 was demonstrated to be up-regulated by HIF-1α in retinal pigment epithelial cells in response to hypoxia, without influencing VEGF secretion.

## Introduction

Alpha-enolase (ENO1) belongs to the enolase family that contains three distinct isoforms, alpha- or non-neuronal enolase, beta- or muscle-specific enolase and gamma- or neuron-specific enolase. ENO1 exists in most eukaryotic organisms and is widely distributed among different human tissues. It functions mainly in the cytoplasm as a key glycolytic enzyme that catalyzes the conversion of 2-phosphoglycerate to phosphoenolpyruvate in the glycolytic metabolic pathway [1]. Glycolysis is a compensatory process of energy metabolism during hypoxia, which is a common pathological condition contributing to diverse diseases like cancer and neovascularization. In several human cancers such as breast cancer, lung adenocarcinoma, glioma, and hepatoma, ENO1 is known to be over-expressed and its expression correlates positively with tumor progression, angiogenesis and venous invasion. This has been validated by not only experimental research but also clinical and pathological characteristics. Hence, ENO1 has been considered as a potential candidate for targeted therapeutic intervention of cancer [2–7].

ENO1 is demonstrated to be up-regulated in the hypoxic cancer and brain cells under the control of the Hypoxia-inducible factor 1-α (HIF-1α) [2, 4–8]. HIF-1α is a master regulator of mammalian oxygen homeostasis. When cells are subjected to hypoxia, HIF-1α serves as a main transcriptional factor activating transcription of genes encoding glycolytic enzymes, vascular endothelial growth factor (VEGF) and other proteins that are important for maintaining oxygen homeostasis via binding to the hypoxia response elements (HRE) in the promoter region of these genes [9, 10]. However, the molecular mechanisms of ENO1 favoring cancer angiogenesis have not yet been clear.

ENO1 is also abundantly expressed in the eye, especially in the ocular epithelial cells where its concentration exceeds any likely requirements for a purely glycolytic role [11]. It is initially identified as τ crystallin expressed in the lens epithelial cells where it fulfills a structural role important for transparency [12, 13]. In the cornea, ENO1 is found at high concentrations in the corneal epithelial cells, making it a known marker for epithelial cell differentiation [14, 15]. Expression of ENO1 is augmented in the limbus and the cornea during epithelial regeneration [16]. On the other hand, decreased levels of ENO1 are demonstrated in keratoconus, a non-inflammatory disorder leading to stromal thinning and epithelial degeneration in the cornea [17, 18]. These studies indicate that ENO1 is important for the function of the eye.

Choroidal neovascularization (CNV) is a leading cause of severe vision loss especially in patients with age-related macular degeneration (AMD). Oxidative injury, resulting from local hypoxia and ischemia in the choroid and the retinal pigment epithelium, has been hypothesized as the key event in the initiation of CNV [19–23]CNV shares the same key contributing factor, hypoxia, with cancer angiogenesis. ENO1, as described before, is an important player versus hypoxic stress. However, compared with cancer angiogenesis, there is very limited understanding of glycolytic enzymes such as ENO1 in CNV. Recent publications identifying anti-α-enolase antibodies in the retina and sera of patients with cancer-associated retinopathy, autoimmune retinopathy and AMD have drawn our attention into the role of ENO1 in CNV [24–26]. In the present study, we investigated the level and regulation of ENO1 in retinal pigment epithelial cells upon hypoxia and its possible contribution to CNV by regulating VEGF secretion.

## Materials and Methods

### Cell culture and anoxia or hypoxia treatment

ARPE-19 is a spontaneously arising human retinal pigment epithelia (RPE) cell line purchased commercially from American Type Culture Collection (Manassas, USA). It has been widely used for *in vitro* studies on the mechanisms of ocular diseases. Cells were cultured at 37°C, 21%O_2_, 5%CO_2_, with complete Dulbecco’s modified Eagle’s medium/F12 (DMEM/F12, Invitrogen, New York, USA) containing 10% fetal bovine serum (FBS, Hyclone, Logan, USA), 100 units/ ml penicillin (Invitrogen) and 100 μg/ ml streptomycin (Invitrogen). Cells between passages 9 to 15 were used for the experiments. 1.5×10^5^ RPE cells/well were seeded in 6-well plates and incubated for 48 hr. Upon reaching about 80–90% confluence, cells were rinsed with and changed to 1ml serum-free Dulbecco’s modified Eagle’s medium (DMEM, Invitrogen,) just before treatment. For anoxic treatment, cells were placed in a sealed anaerobic chamber (Thermo Fisher Scientific, New York, USA) brimmed with 95% N_2_ and 5% CO_2_. For hypoxic treatment, the culture medium was supplemented with 300μM cobalt (II) chloride CoCl_2_ (Sigma, St. Louis, USA), a commonly used hypoxia-mimetic chemical. Twenty-four hr after treatment, the culture medium was collected for Lactate Dehydrogenase assay and Enzyme-linked immunosorbent assay (ELISA). Meanwhile, cell lysates were harvested for Western Blotting.

### Small interfering RNA (siRNA) transfection

ARPE-19 cells were transfected with ON-TARGETplus SMARTpool siRNA (Dharmacon, Thermo Fisher Scientific) via LipofectamineTM 2000 (Invitrogen). Before siRNA transfection, cultures were plated at 1.5×10^5^ RPE cells/well in 6-well plates with antibiotic-free complete DMEM/F-12 medium for 24 hr. Upon 60%-70% cell confluence, 75 nM siHIF and/or 200 nM siENO1 were transfected into cells by mixing with Lipofectamine in OptiMEM^®^I Reduced Serum Medium (Invitrogen) according to the manufacturer’s protocol. The amount of siRNA adopted had been optimized. After 24 hr of incubation, the medium was replaced with 1 ml serum-free DMEM medium and cells were challenged with CoCl_2_-induced hypoxia for another 24 hr. A non-targeting scramble siRNA with RISC-free modification (siGLO Green Transfection Detector, Dharmacon, Thermo Fisher Scientific) was included as the negative control. The FAM-labeled siGLO with the green fluorescence displayed by successfully transfected cells can also help to determine the transfection efficiency.

### Lactate dehydrogenase (LDH) assay

Cytotoxic effects of various treatments to cells were assayed by LDH leakage into the medium, which is an indicator of membrane breakage and cell death [27]. Cultured medium was collected while cell lysates were obtained by addition of 2% Triton X-100 in 1 ml flesh serum-free medium for overnight at 4°C. LDH activities in the supernatant of cell homogenate and the medium were measured using the Cytotoxicity Detection Kit (LDH, Roche Applied Science, Basel, Switzerland) according to the manufacturer’s instruction. Absorbance at 490 nm and 630 nm was measured by a microplate reader (Bio-Tek, Winooski, USA). Readings at 630 nm were subtracted from the readings at 490 nm to allow correction for optical imperfections. Percent leakage of LDH was calculated as {LDH activity in medium/ (LDH activity in medium + LDH activity in cells)} × 100%. Assays were repeated in triplicate.

### Protein collection and Western blotting

After 24 hr of anoxic or hypoxic treatments, cells were rinsed with ice-cold PBS and immediately lysed with RIPA lysis buffer containing protease inhibitor and protein phosphatase inhibitor (Roche Applied Science) on ice. The whole cell lysate was harvested after centrifugation at 13200 rpm for 20 min at 4°C and the supernatant was quickly frozen with liquid nitrogen before storage at -80°C. Protein concentration was determined by Bradford assay (Bio-Rad, California, USA) using bovine serum albumin (BSA, Bio-Rad) as the standard.

The protein levels of HIF-1α and ENO1 were detected by Western blotting. SDS-PAGE was performed at 90V in 4% stacking gel and 120V in 8% separating gel. Proteins were electro-transferred onto a PVDF membrane (Millipore, Billerica, USA) via Mini Trans-blot Cell^®^ (Bio-Rad) at 120V for 1.5 hr on ice. After blocking with 5% skimmed milk in TBST for 1 hr, the membranes were incubated overnight at 4°C with specific primary antibody for HIF-1α (monoclonal, mouse, Thermo Scientific) or ENO1 (monoclonal, rabbit, Abcam, Cambridge, UK). Due to the large difference in the molecular mass of HIF-1α and ENO1, β-actin (mouse, Chemicon, Tokyo, Japan) or p84 (mouse, Genetex, Irvine, USA) was chosen, respectively as internal loading controls. Immunoreactivities were visualized with HRP-conjugated secondary antibodies (Vector Laboratories, Burlingame, USA) and ECL Chemiluminscent Substrate (GE Healthcare, Little Chalfont, UK). The signals were captured using X-ray films (Fuji, Tokyo, Japan) or a CCD camera-based imager (My ECL Imager, Thermo Fisher Scientific). Integrated intensities of target bands on the Western blot images were determined by densitometric image analysis using Image J software (National Institute of Health, Bethesda, USA). The band intensity of the target protein was further normalized against β-actin or p84. Fold changes of protein expressions were calculated versus the normal control level.

### Enzyme-linked immunosorbent assay (ELISA) of VEGF

Culture medium was assayed for VEGF concentration using the Quantikine^®^ Human VEGF Immunoassay Kit (R&D Systems, Minneapolis, USA) according to the manufacturer’s instructions. Cell culture medium was collected and supplemented with 2% fetal bovine serum (Hyclone) before storage at -20°C for maintaining the stability of VEGF. All reagents and standards were freshly prepared and added during the assay as instructed by the manufacturer. The concentration of VEGF was measured by the color intensity of solutions using a microplate reader (Bio-Tek) at 450 nm and 570 nm, respectively. Readings at 570 nm were subtracted from the readings at 450 nm to allow correction for optical imperfections. VEGF concentrations were obtained by comparing the corresponding readings with those of the standard curve using known concentrations of VEGF. Assays were repeated in duplicate.

### Statistical analysis

Statistical analyses were performed using the GraphPad Prism 6 software (GraphPad Software, La Jolla, USA). Data presented as mean ± S.D. were analyzed by paired t-test (2 groups) or one-way ANOVA (> 2 groups) followed by Bonferroni’ s post test [28]. The post tests were performed only when the means of the groups were significantly different (p<0.05).

## Results

### No observable cell death in ARPE-19 cells after 24 hr of anoxia or CoCl_2_-induced hypoxia

**Fig 1:**
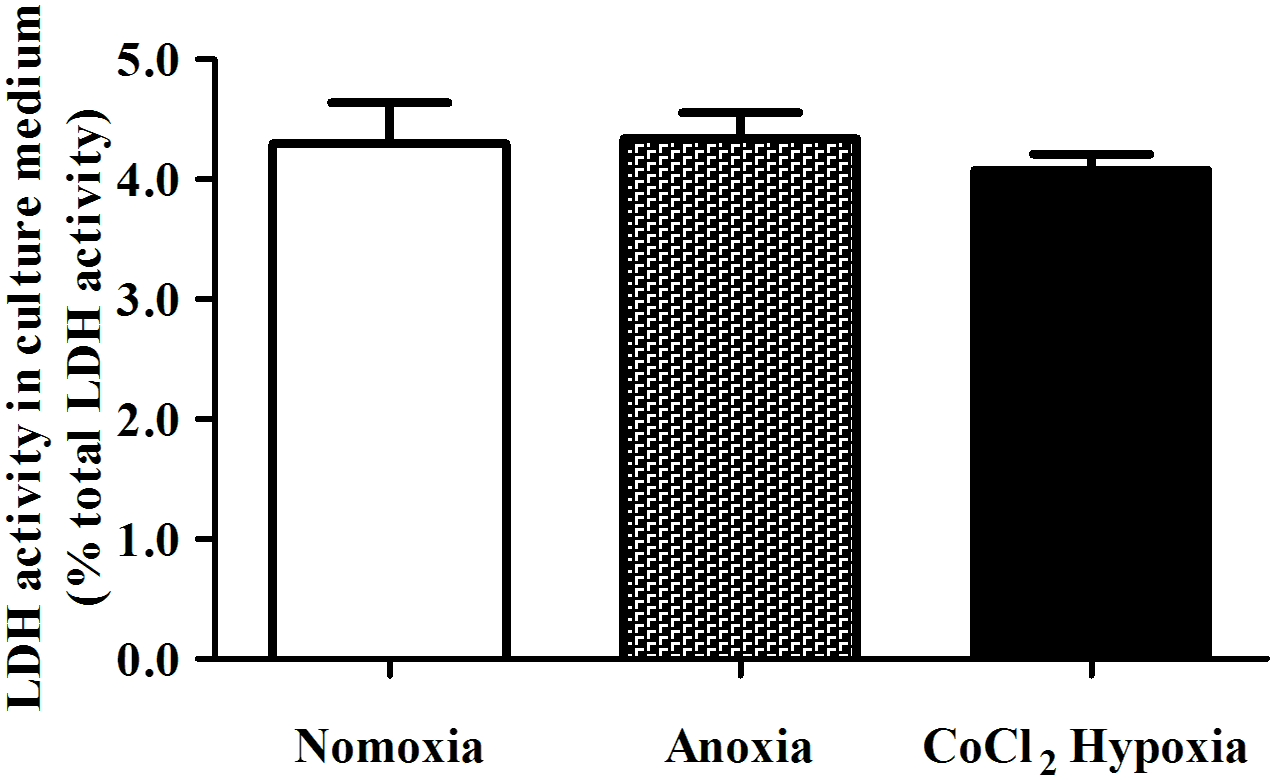
LDH analysis of ARPE-19 cell death after 24 hr of anoxia or CoCl_2_-induced hypoxia. Serum-starved ARPE-19 cells were challenged with anoxia or CoCl_2_-induced hypoxia. LDH activities in the culture medium were detected after 24 hr of treatment. No significant differences in the LDH activities were observed in cells under normoxic, anoxic and CoCl_2_-induced hypoxic conditions. This implied that no remarkable cell death (LDH release less than 5%) resulted from the anoxic or hypoxic stress in the cultured ARPE-19 cells. (n = 4).

The overall cellular damage displayed by ARPE-19 cells in response to anoxia or hypoxia was analyzed by LDH cytotoxicity assay. LDH is a cytoplasmic enzyme that is rapidly released into the surrounding medium upon cell damage. As a result, the activity of LDH in the medium is proportional to the extent of cell death; the higher LDH activity indicates more cell damage [27]. Interestingly, after 24 hr of anoxia or hypoxia, there appeared to be no observable cell death in either anoxic-treated or CoCl_2_-treated ARPE-19 cells when compared with the normoxic cells (Fig 1), a result similar to previous findings in cultured ARPE-19 cells upon 24 hr of hypoxic treatment [22]. Morphological analysis by light microscopy also revealed no detectable cell death (data not shown).

### Induction of HIF-1α and ENO1 in ARPE-19 cells upon 24 hr of anoxia or hypoxia challenge

**Fig 2:**
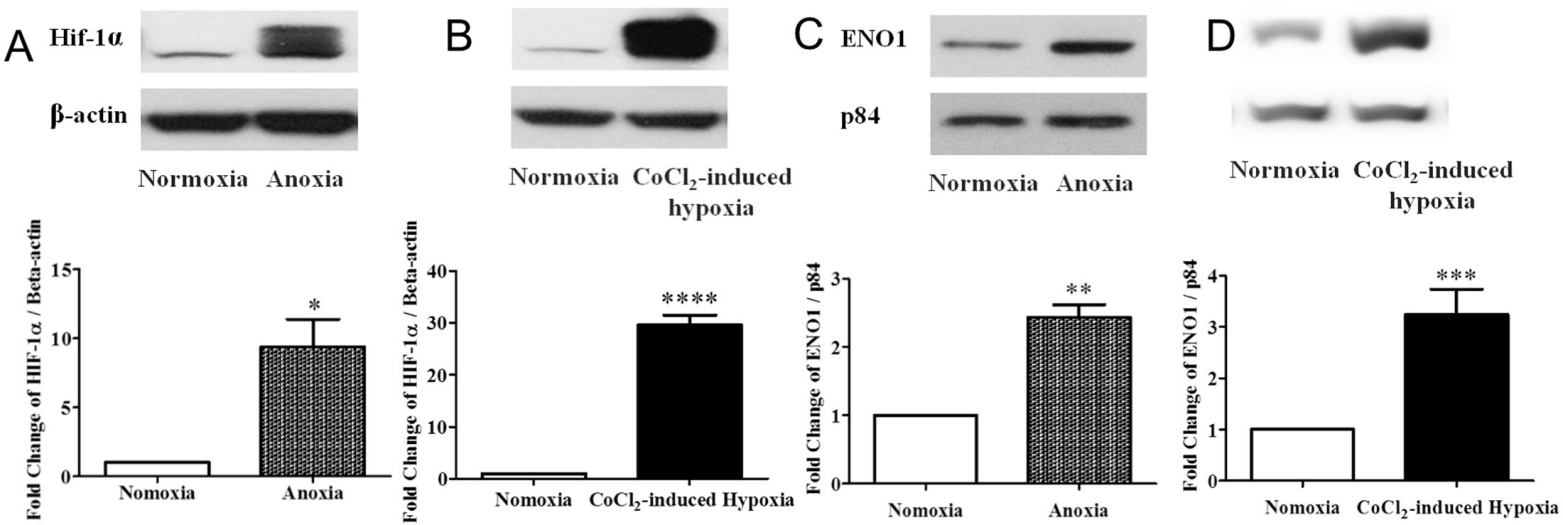
Changes in HIF-1α and ENO1 levels in ARPE-19 cells after 24 hr of anoxic or hypoxic treatment. ARPE-19 cells were cultured with deoxygenated DMEM medium or supplemented with 300μM CoCl_2_ for 24 hr. (A. B) Expression of HIF-1α (116kDa) and (C, D) ENO1 (46kDa) were assayed by Western blotting. β-actin (42kDa) and p84 (84kDa) were used, respectively as the internal control for equal loading of proteins. In ARPE-19 cells, HIF-1α protein level was barely detectable under normoxia. (A) Upon 24 hr of anoxia, HIF-1α expression rose significantly (n = 3, *p < 0.05 versus normoxia). (B) CoCl_2_-induced hypoxia also successfully increased HIF-1α protein levels in ARPE-19 cells (n = 6, ****p<0.001 versus normoxia). In contrast to the normoxic control, the protein level of ENO1 increased significantly under both treatment conditions. (C) It approximately doubled after 24 hr of anoxia (n = 3, **p<0.01 versus normoxia) while it was tripled in the CoCl_2_-treated cells. (n = 6, ***p<0.001 versus normoxia) (D).

HIF-1α protein was barely detectable in ARPE-19 cells under normoxia (Fig 2A & 2B). After a 24-hr of either anoxia or CoCl_2_-induced hypoxia, HIF-1α levels were significantly elevated (Fig 2A & 2B). Moreover, the increase in HIF-1α protein level in ARPE-19 cells under CoCl_2_ challenge (~30 times) (Fig 2B) was much larger than under anoxia (~8 times) (Fig 2A). These results indicated that HIF-1α was successfully induced by 24 hr of either treatment, with the CoCl_2_-induced hypoxia causing a much stronger and more stable response from ARPE-19 cells than the anoxic treatment.

On the other hand, ENO1 protein was highly expressed in ARPE-19 cells under normal condition (Fig 2C). Significant increases in ENO1 levels were also found in ARPE-19 cells when exposed for 24 hr to either anoxia (Fig 2C) or CoCl_2_-induced hypoxia (Fig 2D). Hence, we demonstrated that the expression of ENO1 is increased in ARPE-19 cells along with HIF-1α after anoxia or CoCl_2_-induced hypoxia. Since the effect of CoCl_2_-induced hypoxia was more stable and remarkable, only CoCl_2_ treatment was used in the rest of our experiments.

### Transfection efficiency of siRNA in ARPE-19 cells

**Fig 3:**
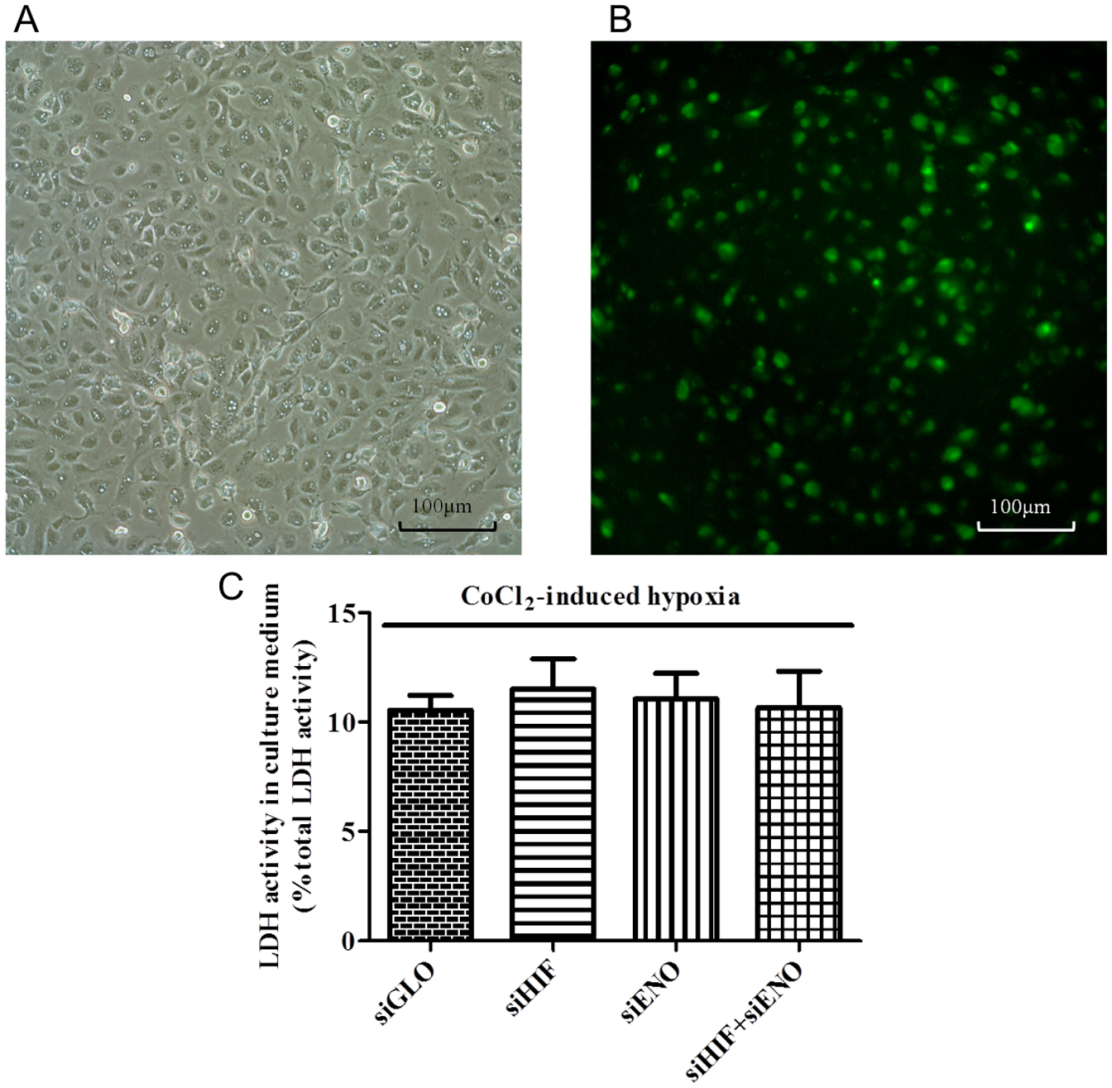
(A, B) Representative photomicrographs of ARPE-19 cells at 48 hr of transfection with FAM-labeled siGLO RISC-free siRNA. RPE cells were transfected with scramble control siRNA (siGLO) for 24 hr and subsequently cultured with 300μM CoCl_2_ for another 24 hr. Cell images were captured at 48 hr under bright field (A) and fluorescence field with a FITC filter (B) at 200X magnification. Cells displayed green fluorescence, indicating that they were transfected with FAM-labeled RISC-independent siRNA (siGLO), which was used as both the scramble control and the transfection indicator. Under our experimental condition, over 60% of cells showed observable fluorescence suggesting successful transfection after 48 hr. (C) LDH analysis of transfected ARPE-19 cells under 24 hr of CoCl_2_-induced hypoxia. ARPE-19 cells were transfected with scramble control siRNA (siGLO), HIF-1α siRNA (siHIF), ENO1 siRNA (siENO), and co-transfected with both HIF-1α siRNA and ENO1 siRNA (siHIF + siENO) for 24 hr, followed by another 24 hr of CoCl_2_-induced hypoxia before LDH assay. Compared with the control group, lack of changes in the LDH activities were observed in the cells transfected with different targeting siRNA under CoCl_2_ challenge. All cell survival rates were over 85%. (n = 4).

To further study the regulation and role of ENO1 in ARPE-19 cells in response to oxygen depletion, siRNA was applied to mediate its down-regulation. Transfection efficiency of the siRNA is an important factor for effective down-regulation of the target mRNA and protein. The optimal siRNA transfection conditions in our study were determined using the FAM-labeled RISC-independent control siRNA (siGLO) as the transfection indicator. After transfecting the ARPE-19 cells with 75nM siGLO for 48 hr, more than 60% of the RPE cells displayed green fluorescence (Fig 3A & 3B). Meanwhile, the survival rates of transfected cells were all over 85%. Single or co-transfection of siRNAs for HIF-1α and ENO1 had no effect on ARPE-19 cell toxicity as assessed by LDH leakage assay (Fig 3C), similar to previous reports [3]. Both conditions described above ensured that the majority of ARPE-19 cells were successfully transfected with siRNA and stayed alive in our experiments. There was also little/no difference in total protein content among various transfected cultures, as all siRNA transfection did not cause cell damage or reduce protein synthesis.

### Decreased ENO1 expression in HIF-1α-diminished ARPE-19 cell under CoCl_2_-induced hypoxia

**Fig 4:**
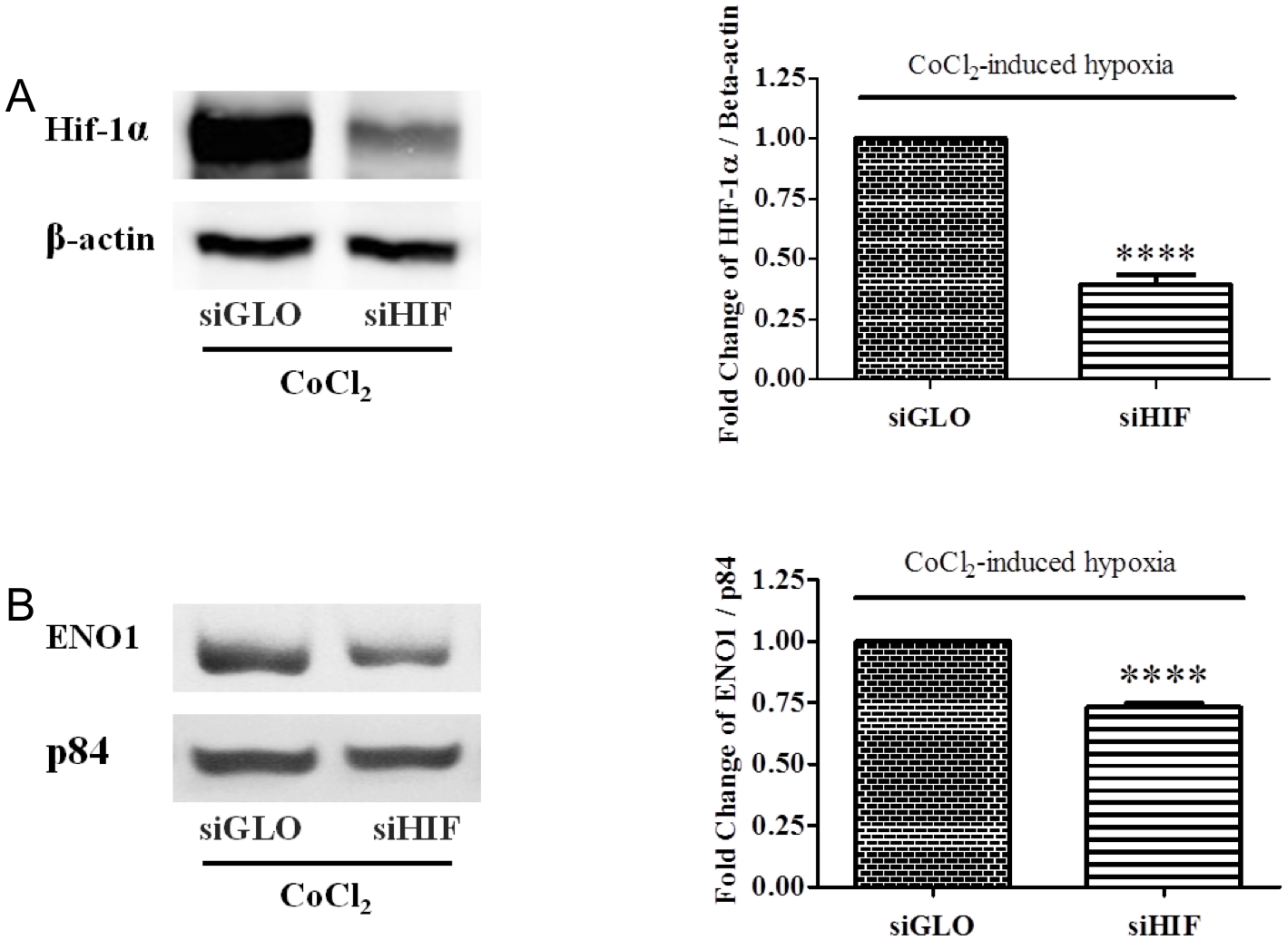
Changes of HIF-1α & ENO1 levels in siHIF-transfected ARPE-19 cells at 24 hr of hypoxia. ARPE cells were transfected with scramble control (siGLO) or HIF-1α siRNA (siHIF) for 24 hr, and cultured with 300μM CoCl_2_ for another 24 hr. Expressions of HIF-1α and ENO1 were assayed by Western blotting. β-actin and p84 were used as the internal controls, respectively. (A) Upon transfection with siHIF, the HIF-1α level under hypoxia condition was clearly reduced when compared with the control cells transfected with siGLO (n = 6, ****p< 0.0001). (B) The expression of ENO1 in the hypoxic ARPE-19 cells displayed a decrease by about 30% upon HIF-1α diminished (n = 6, ****p< 0.0001).

Knockdown of HIF-1α in the hypoxic ARPE-19 cells was performed using HIF-1α siRNA (siHIF). The result of Western blotting showed a significant down-regulation of HIF-1α protein level in the siHIF-transfected ARPE-19 cells under hypoxic stress (Fig 4A). Meanwhile, reduction of ENO1 expression was also observed when HIF-1α was successfully knocked down. ENO1 protein level was decreased by about 30% in the HIF-1α-diminished ARPE-19 cell when compared with the negative control cells (Fig 4B).

### siRNA-mediated knockdown and double-knockdown of ENO1 in ARPE-19 cell under CoCl_2_-induced hypoxia

**Fig 5:**
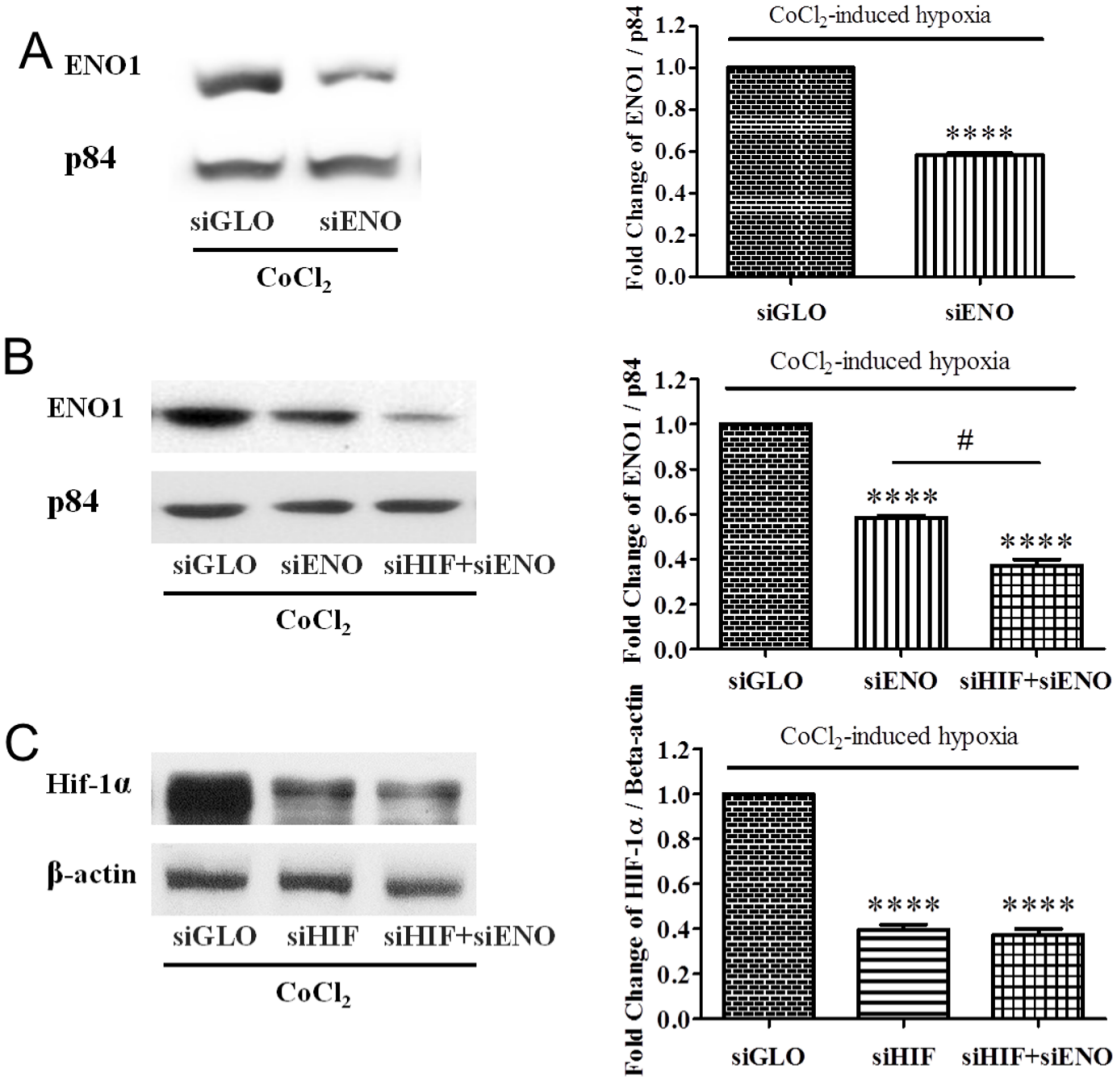
Changes of ENO1 and HIF-1α levels in siENO-transfected ARPE-19 cells at 24 hr of hypoxia. (A) ARPE-19 cells were transfected with siENO for 48 hr. In the meantime, cells were exposed to CoCl_2_-induced hypoxia for the last 24 hr. The protein level of ENO1 managed to be reduced by about 40% in the hypoxic ARPE-19 cells. (B) ARPE cells were (co-)transfected with scramble control siRNA (siGLO), HIF-1α siRNA (siHIF) and/or ENO1 siRNA (siENO) for 24 hr, respectively and cultured with 300μM CoCl_2_ for another 24 hr. ENO1 protein was successfully suppressed by transfecting with siENO (~40%), the expression of which was further knocked down by co-transfecting with both siHIF and siENO (~60%) (n = 4, ****p<0.0001 versus siGLO respectively; # p<0.0001). (C) Under hypoxia, HIF-1α protein was significantly decreased (~60%) in ARPE-19 cells with the transfection of siHIF. Co-transfection with siHIF and siENO did not further down-regulate the protein level of HIF-1α, which was nearly the same as that in the siHIF-transfected cells (n = 4, ****p<0.0001 versus siGLO respectively). (c, d).

In order to further investigate the role of ENO1 in hypoxia, ENO1 siRNA (siENO) was used to knock down ENO1 in the hypoxic ARPE-19 cells. As ENO1 is highly expressed in the retinal pigment epithelial cells even under normoxia, the amount of siRNA was modified and optimized. The protein level of ENO1 in cells transfected with 200 nM siENO was decreased by about 40% under hypoxia (Fig 5A), which was similar to the published results [3]. Co-transfection with siHIF and siENO managed to further decrease ENO1 expressions in the hypoxic ARPE-19 cells (Fig 5B). Conversely, knockdown of ENO1 had little/no effect on the induction of HIF-1α in ARPE-19 cells under hypoxia (Fig 5C). This result is similar to the previously reported results in cancer research [4] and suggested that ENO1 is the downstream product and does not affect HIF-1α expression in ARPE-19 cells.

### Changes in VEGF expression in ENO1-knockdown or HIF-1α & ENO1-double knockdown ARPE-19 cells under hypoxia

**Fig 6:**
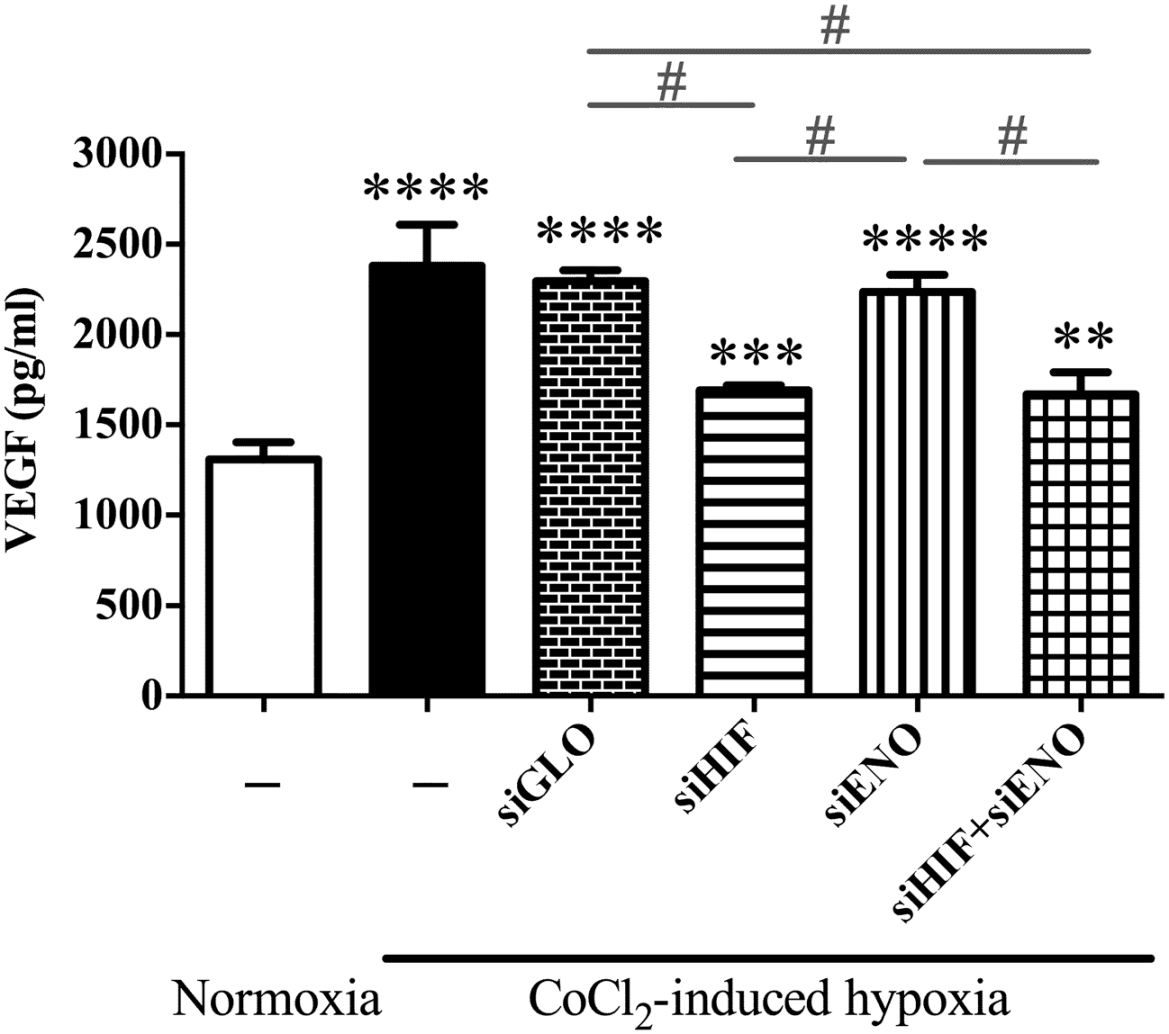
VEGF secretion in ARPE-19 cells at 24 hr of normoxia or hypoxic treatment. Culture medium was assayed for VEGF secretion by ELISA. (Lanes 1&2) VEGF secretion in ARPE-19 cells was strongly induced in response to hypoxia. (Lanes 3&4) Down-regulation of HIF-1α markedly but not completely alleviated VEGF level in ARPE-19 cells. The level of VEGF secretion in HIF-1α-diminished ARPE 19 cells was still higher than that under normoxia. (Lanes 3&5) VEGF secretion in ENO1-knockdown ARPE 19 cells remained nearly the same as siGLO-transfected cells under hypoxia. (Lanes 3, 4&5) Double-knockdown of HIF-1α and ENO1 significantly down-regulated VEGF level when compared with that in the singly ENO1-diminished cells, while it appeared unchanged when compared with that in the HIF-1α knockdown cells. (n = 4, **p<0.01, ***p<0.001, ****p<0.0001, versus normoxia respectively; #p<0.0001).

ELISA results demonstrated that HIF-1α also controlled the up-regulation of VEGF in the hypoxic RPE cells. Expression of VEGF was markedly augmented in ARPE-19 cells in response to hypoxia (Fig 6 lanes 2&3), along with HIF-1α (Fig 2B) and ENO1 (Fig 2D). Decreasing HIF-1α expression by siRNA in the hypoxic ARPE-19 cells managed to down-regulate the VEGF secretion (Fig 6 lane 4) as well as ENO1 expression (Fig 4B). However, VEGF was only partially suppressed in the HIF-1α-diminished ARPE-19 cells under hypoxia challenge and its level was still a little higher than that in normal condition (Fig 6 lanes 1&4). Interestingly, knockdown of ENO1 turned out not to help alleviate VEGF secretion upon hypoxia. The level of VEGF in siENO-transfected ARPE 19 cells remained nearly the same as that in the normal and siGLO-transfected cells under hypoxia (Fig 6 lane 5). Moreover, double-knockdown of HIF-1α and ENO1 could not further down-regulate VEGF secretion, which was very similar to that in the HIF-1α-diminished cells (Fig 6 lanes 4&6).

## Discussion

HIF-1α is the master transcriptional factor of various angiogenic factors (e.g. VEGF) involved in CNV [29]. It is hardly stable and detectable under normoxia. However, external stress such as depletion of oxygen would inhibit the degradation of HIF-1α [30, 31]. Although either anoxia or CoCl_2_–induced hypoxia could significantly increase HIF-1α expression in ARPE-19 cells, the respective levels of HIF-1α up-regulation seemed different. According to our results, HIF-1α protein induction appeared to be more prominent upon 24 hr of CoCl_2_–induced hypoxia in contrast to anoxia. In fact, CoCl_2_-induced hypoxia appeared to be more pronounced and stable, reducing the degradation of HIF-1α in a dose-dependent response. Therefore, CoCl_2_-induced hypoxia was chosen as the treatment in later experiments.

ENO1 has been identified as a hypoxic stress protein in vascular endothelial cells, where its protein level rose to nearly three fold at 24 hr of anoxic exposure [32], similar to our results obtained from retinal pigment epithelial cells. In previous proteomic studies, ENO1 was found in ARPE-19 cells [33] and changes in ENO1 expression were observed during chick retinogenesis [34]. Herein, we are the first to demonstrate that ENO1 is one of the distinct proteins that is up-regulated in the retinal pigment epithelial cells by hypoxia, in conformity with the findings in cancer angiogenesis.

Analysis of ENO1 gene has revealed that ENO1 promoter contains a hypoxia responsive element (HRE). In response to hypoxia, accumulated HIF-1α activates the transcription of ENO1 by recognizing HRE, as is the case for VEGF activation [2, 9, 35–37]. The mRNA and protein levels of ENO1 and VEGF can therefore be induced by HIF-1α in hypoxic retinal pigment epithelial cells.

VEGF is a potent pro-angiogenic factor produced by retinal pigment epithelial cells and a well-known HIF-1-dependent factor. We showed that ENO1 level did not change VEGF expression in retinal pigment epithelial cells, indicating that either ENO1 or VEGF is independently induced by hypoxia and ENO1 may not play a role in regulating VEGF secretion in retinal pigment epithelial cells. Since overexpression of ENO1 has been demonstrated to promote proliferation and migration of endothelial cells, it is likely that pathways other than VEGF may be involved.

The potential role and mechanism of ENO1 in promoting retinal angiogenesis has yet to be understood. Some ENO1-regulated proteins have been identified using two-dimensional electrophoresis, including C1 esterase, pyruvate kinase, and FR-β in our preliminary work [38]. Further studies in examining how knockdown of ENO1 affects the migration and proliferation of endothelial cells will help to decipher the involvement of ENO1 in retinal angiogenesis. The effect of ENO1 alteration on VEGF receptor expression on endothelial cells may also help to clarify this point.

## Conclusion

ENO1 exists in the retinal pigment epithelium at high concentration. When the retinal pigment epithelial cells and the choroid are subjected to pathological hypoxia or ischemia, expression of ENO1 is up-regulated in the retinal pigment epithelial cells mediated by HIF-1α, which also increases VEGF secretion in the retinal pigment epithelium. Nevertheless, ENO1 has no effect on influencing VEGF expression in the retinal pigment epithelial cells, which means that they are independently regulated by HIF-1α without mutual interference. Together with VEGF up-regulation, over-expression of ENO1 in the retinal pigment epithelium in response to hypoxia may suggest a potential positive role in the development of CNV.
